# Determining the Association between Language and Cognitive Tests in Poststroke Aphasia

**DOI:** 10.3389/fneur.2017.00149

**Published:** 2017-05-05

**Authors:** Kylie J. Wall, Toby B. Cumming, David A. Copland

**Affiliations:** ^1^Centre for Clinical Research, School of Health and Rehabilitation Sciences, The University of Queensland, Brisbane, QLD, Australia; ^2^The Florey Institute for Neuroscience and Mental Health, University of Melbourne, Melbourne, VIC, Australia

**Keywords:** aphasia, cognition, cognitive impairments, stroke, neuropsychological tests, pen-and-paper tests

## Abstract

**Background:**

Individuals with aphasia are often excluded from studies exploring poststroke cognition because so many of the standard cognitive assessments rely on language ability. Our primary objective was to examine the association between performance on cognitive tests and performance on comprehension and naming tests in poststroke aphasia. Second, we aimed to determine the association between language performance and a real-life measure of cognition (Kettle Test). Third, we explored the feasibility of administering cognitive tests in aphasia.

**Methods:**

Thirty-six participants with poststroke aphasia and 32 controls were assessed on a battery of pen-and-paper cognitive tests recommended in stroke. Auditory comprehension was measured using the Comprehensive Aphasia Test and naming was measured using the Boston Naming Test. Twenty-two community dwelling participants with aphasia and controls were also asked to complete the Kettle Test. Multiple linear regressions were used to explore the relationship between language performance and performance on the cognitive tests. Feasibility was determined by quantifying missing data.

**Results:**

The cognitive tests with the highest variance accounted for by auditory comprehension and naming were animal fluency (*R*^2^ = 0.67, *R*^2^ = 0.78) and the Hopkins Verbal Learning Test (recognition discrimination index) (*R*^2^ = 0.65, *R*^2^ = 0.78). All cognitive tests were significantly associated with auditory comprehension and naming, except for the Star Cancellation Test and the Kettle Test. Thirty-three percent of participants with aphasia were unable to complete all the cognitive tests.

**Conclusion:**

Language and non-linguistic cognitive processes are often interrelated. Most pen-and-paper cognitive tests were significantly associated with both auditory comprehension and naming, even in tests that do not require a verbal response. Language performance was not significantly associated with a real-life cognitive performance measure. Task instructions, stimuli, and responses for completion need to be tailored for individuals with aphasia to minimize the influence of language deficits when testing non-linguistic cognitive performance.

## Introduction

Up to 30% of stroke survivors experience difficulty with receptive and expressive language—called aphasia (1). There is an assumed relationship between language and non-linguistic cognitive performance in poststroke aphasia, but the nature and management of this relationship is poorly understood. Studies show that impaired executive skills, working memory, and attention can adversely influence aphasia rehabilitation outcomes (2–4), and cognitive performance may predict aphasia recovery better than language performance (5). El Hachioui et al. (6) explored cognitive deficits in aphasia during the first year poststroke and the association with functional outcome. Participants with persisting aphasia had poorer cognitive performance, poorer functional outcome, and they were more depressed compared to participants with resolved aphasia. To optimize aphasia therapy, clinicians need to measure linguistic and non-linguistic performance to ensure all aspects of cognitive impairments are considered.

International guidelines recommend that all stroke survivors should be screened for cognitive impairments using valid and reliable tools, and comprehensive neuropsychological testing should be undertaken for those that fail screening (7–9). Pen-and-paper screening tools and assessments are used more frequently than alternative methods for assessing cognition poststroke (10, 11). Such tools are often linguistically loaded, and aphasic deficits may confound non-linguistic cognitive performance (12, 13). Consequently, patients with aphasia are often excluded from studies validating cognitive assessments and exploring cognitive outcomes in stroke (10, 14, 15).

A reliance on verbal response is an obvious barrier to obtaining accurate measures of non-linguistic cognitive performance in aphasia. For example, verbal fluency is task often used in standard language assessments (16), but it is also used to measure executive skills in stroke (17). This highly language-dependent task (18) is unlikely to accurately represent executive skills in aphasia. Yet, eliminating verbal responses may not resolve language deficits confounding non-linguistic cognitive performance in aphasia. Comprehension deficits associated with aphasia may also confound results. Cognitive tests are often complex, with detailed instructions requiring sophisticated comprehension skills to understand the tasks (19). Increased syntactical complexity negatively influences comprehension in aphasia (20), and the linguistic complexity of instructions needs consideration in this stroke subgroup.

To quantify the association between language performance and cognitive tests without a verbal response, Fucetola et al. (21) explored how much variance in the non-verbal subtests from the Wechsler Adult Intelligence Scale-III (block design, matrix reasoning, and picture arrangement) and Wechsler Memory Scale-III (spatial span) was accounted for by auditory comprehension and oral expression in aphasia. Auditory comprehension accounted for 41% of the total variance (*p* < 0.001), whereas no significant relationship was found with naming performance. This study suggests that non-verbal cognitive performance is related to auditory comprehension severity, but 59% of the variance remains unexplained.

Cognitive tests vary in the cognitive domain being tested, the task complexity, the delivery of instructions, and the responses needed for completion. There has been no systematic analysis of the relationship between language performance in poststroke aphasia (naming and comprehension) and performance on a broad range of widely used neuropsychological tasks. Exploring the potential variability in the association between language and scores on cognitive tests (including an everyday real-life measure of cognition, such as making a hot drink) in aphasia is necessary to better inform clinical practice.

Our primary objective was to examine the association between performance on cognitive tests and assessments of comprehension and naming in poststroke aphasia. Our second aim was to determine the association between auditory comprehension and naming performance and a validated real-life cognitive performance assessment in aphasia and controls. Our last aim was to determine the feasibility of all cognitive tests used by quantifying missing data in patients with aphasia compared to controls.

## Participants and Methods

### Participants

Thirty-six participants with poststroke aphasia and 32 controls were recruited from three Brisbane Hospitals, the Communication Registry at The University of Queensland, community posters, social groups, and newsletters. Participants with aphasia had diagnostic imaging evidence of stroke (or a clinical diagnosis if imaging was unavailable) and a diagnosis of aphasia according to the Comprehensive Aphasia Test (CAT) (using auditory comprehension subtests’ cutoff scores) (16) or the Language Screening Test (cutoff < 15) (22). Patients were excluded if they: (1) had visual and hearing impairments that impeded testing; (2) needed an interpreter to participate if English was their second language; or (3) were too medically unwell. The included control participants passed a mood screen (The Patient Health Questionnaire) (23) to eliminate the potential influence of depression on cognitive performance (24, 25). Controls were excluded if they had a history of neurological disease or acquired injury, or if they needed an interpreter to participate if English was their second language.

### Assessments

Demographic data collected included age, sex, education level, handedness, time poststroke, and clinical setting. We did not report localization of stroke lesion(s) because detailed neurological data could not be sourced for all community participants.

Language performance and severity of aphasia were assessed using the CAT (16) (auditory comprehension total score) and the 15-item abbreviated Boston Naming Test (26). The Boston Naming Test is one of the most widely used standardized aphasia measures in clinical practice (27). The 15-item abbreviated Boston Naming Test strongly correlates with the full Boston Naming Test (*r* = 0.93) (28), and it was recommended as part of neuropsychological testing for stroke survivors (17). Fifty percent of stroke survivors experience fatigue irrespective of time poststroke (29). The practicality of testing individuals with fatigue was considered in selecting our battery.

Our battery of pen-and-paper neuropsychological tests has been validated in stroke. The battery included as follows.

#### Star Cancellation (30)

A visual neglect test that includes small stars on an A4 sheet with visual distractors (large stars and letters). Participants are provided with a visual demonstration, along with brief verbal instruction, to cross out all the small stars using a pen.

#### The Brixton Spatial Anticipation Test (31)

An executive function test with a 56-page stimulus booklet. It is a visuospatial sequencing task with rule changes where participants are required to detect rules in sequences of stimuli. Each page contains 2 rows of 5 circles, numbered from 1 to 10. On each page, a single circle is colored blue, and the position of the blue circle changes from one page to the next, based on a series of patterns. Participants are provided with lengthy verbal instructions and a practice. The examiner clarifies understanding. Participants are required to point to where they predicted the filled circle will be on the following page, based on the pattern inferred from the previous page.

#### Trail Making Test (Parts A and B) (32)

Part A is often used to test attention. Participants are verbally instructed to connect circles numbered 1–25 in correct order as quickly as possible using a pen. Part B is an executive task where participants are verbally instructed to connect numbered and lettered circles in correct alternating order (i.e., 1-A-2-B, etc.) as quickly as possible. Both parts have practice trials for familiarization.

#### Digit Span Test (Forwards and Backwards) (33)

The forwards test is used to measure verbal short-term memory. Participants are verbally instructed to repeat strings of numbers of increasing length. The backwards test is used to measure verbal working memory and executive skills. Participants are presented with more number of strings, and they are verbally instructed to recall each number string in reverse order.

#### Hopkins Verbal Learning Test (HVLT)-Revised (34)

Hopkins Verbal Learning Test-Revised is used to assess verbal memory. The examiner reads a list of 12 words (from 3 taxonomic categories). Participants are instructed to try to remember, and verbally repeat, as many words as possible from the list. The examiner then reads the same list twice more, with recall each time. The immediate recall score is the total number of words recalled over these three trials. Subsequently, the participants are asked to recall the word list 20–25 min later (delayed recall). A retention score is calculated to determine the percentage of words retained (delayed recall as a percentage of the best immediate recall from trial 2 or 3). This is followed by a forced-choice recognition test [recognition discrimination index (RDI)], where 12 target words from the learning trials are included with 12 distractor words (six semantically related and six semantically unrelated). Participants are instructed to provide a yes/no response.

#### Rey Complex Figure (Copy, Immediate, and Delayed Recall) (35)

Rey Complex Figure (copy, immediate, and delayed recall) is used to assess visuospatial, visual memory, and executive skills. Participants are provided with a pen and paper and asked to reproduce the complex figure. The stimulus figure and reproduction are then removed. After a 5 min delay, the participants are verbally instructed to reproduce the figure from memory. Then, after a 20–30 min delay, the participants are instructed to reproduce the figure from memory again.

#### Animal Fluency (36)

A verbal fluency task where participants are verbally instructed to name as many different animals as possible within a minute. While fluency tasks (such as animal fluency) undoubtedly include facets of executive function in planning search and retrieval, they are predominantly a reflection of language skills (18).

#### Kettle Test (37)

Kettle Test is a real-life everyday performance measure designed to detect cognitive processes needed for independent community living. Observations are rated on 13 distinct steps to complete the hot drink making task and guidelines for cueing are provided. The participants are scored according to the degree of cueing needed to complete the individual steps (0–4). Total scores range from 0 to 52, with higher scores indicating more assistance.

### Statistical Analysis

The relationships between auditory comprehension, naming, and cognitive function were tested using separate multivariate linear regressions (controlling for age and education) for each cognitive test. To determine the distinct effects of auditory comprehension and naming, the independent variables were entered into different models. Demographic variables included in the models were years of education and age. If assumptions were not met to perform the multiple linear regressions, logistic regressions were used. To explore the feasibility of performing cognitive tests in aphasia compared to controls, we recorded reasons for missing data and the frequency for each individual test. All analyses were performed with Stata 14 software.

## Results

The characteristics of the 36 participants with poststroke aphasia and 32 controls are shown in Table 1. Of the 36 participants with aphasia, 22 community dwelling participants and the controls were also asked to complete the Kettle Test. The Kettle Test was not performed in the acute phase of stroke due to practical restrictions on the ward.

**Table 1:** **Characteristics of the aphasia and control groups**.

	Aphasia	Controls
Age in years, mean ± SD	70.1 ± 9.0	67.3 ± 12.3
Sex, *n* (%)		
Female	12 (33)	17 (53)
Male	24 (67)	15 (47)
Handedness, *n* (%)		
Right-handed	34 (94)	30 (85.7)
Left-handed	2 (5.5)	2 (6.3)
Ambidextrous	1 (2.7)	0
Education in years, mean ± SD	11.0 ± 2.6	15.1 ± 3.4
Premorbid neurological disease/injury (*n*)	3	–
Time poststroke, mean ± SD by clinical setting		–
Acute setting (*n* = 12)	9.2 ± 13.2 days	–
Inpatient rehabilitation (*n* = 2)	23.5 ± 11.5 days	–
Community dwelling (*n* = 22)	6.35 ± 5.2 years	–

The severity of auditory comprehension and naming impairments in the aphasia group ranged from very severe to mild language deficits. Total scores for auditory comprehension ranged from 5/66 to 63/66 (median = 53, interquartile range = 29–58) as measured by the CAT. The results from the Boston Naming Test ranged from 0/15 to 15/15 (median = 10, interquartile range = 1–12). Control participants completed all tests, while 33% (*n* = 12) of participants with aphasia had missing data. All participants completed the auditory comprehension and naming tasks. There were a total of 32 missed cognitive test scores.

**Figure 1:**
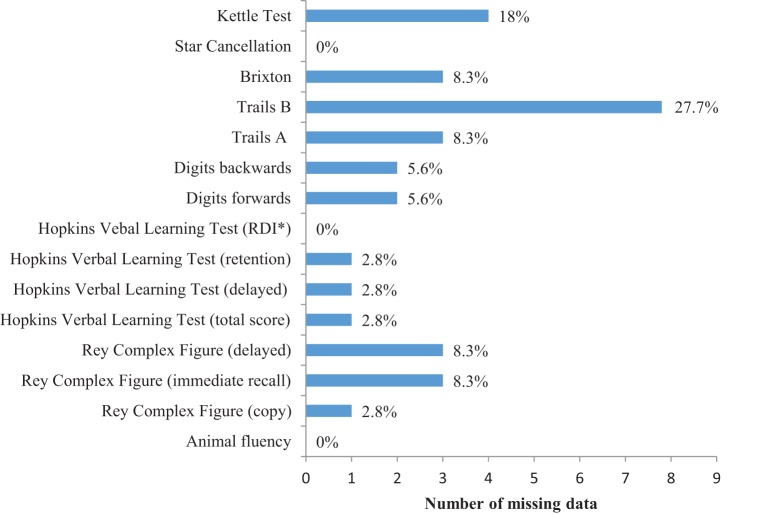
**Number and frequency of missing data by cognitive tests in aphasia**. *RDI, recognition discrimination index.

Figure 1 shows the number and frequency of missing data for the cognitive tests. The Trail Making Test (part B) had more missing data than any other test (28%). The non-verbal cognitive tests had more missing data compared to the tests that required a verbal response. For example, verbal fluency (0%) and the HVLT (0–2.8%) compared to the Brixton (8.3%) and the Rey immediate and delayed recall (8.3%). Reasons for missing data in the pen-and-paper tests were (1) refusal to attempt test (*n* = 3 participants), (2) incomplete due to task complexity (*n* = 3 participants), (3) unable to understand instructions (*n* = 3 participants), and (4) incomplete due to difficulty using a pen (*n* = 2 participants). Four of the 22 community dwelling participants with aphasia (15%) had missing data for the Kettle Test due to upper and lower limb hemiparesis. Participants with missing data had more severe auditory comprehension deficits (median = 27.5, interquartile range = 25.0–49.0) and more severe naming deficits (median = 1, interquartile range = 0–7.5), compared to participants without missing data (auditory comprehension median = 53, interquartile range = 45.8–58.0; naming median = 6.5–13.5, interquartile range = 7.0). The clinical setting did not influence missing data, where there was an equal distribution of participants in the acute versus community setting.

Table 2 shows the descriptive statistics for the language and cognitive tests. As expected, there was minimal variance in the auditory comprehension and the Boston Naming Test scores of the control group, and therefore no regressions associating language and cognitive performance were run in this group. The data for the regressions were sourced only from the participants with aphasia. We conducted a pairwise regression between the independent variables (auditory comprehension and naming), and confirmed that they were too closely related (pairwise correlation = 0.86) to be included in the same regression model.

**Table 2:** **Descriptive statistics of the language and cognitive tests**.

Test	Aphasia group			Controls		
	Mean (SD)	Median	Range	Mean (SD)	Median	Range
Auditory comprehension	46.1 (15.5)	52.0	5–63	61.4 (3.2)	62.0	55–66
Boston Naming Test	8.2 (5.3)	9.5	0–15	13.8 (1.2)	14.0	11–15
Kettle Test	4.6 (4.0)	4.0	0–15	1.5 (1.6)	1.0	0–5
Star Cancellation	51.6 (6.3)	54.0	24–54	53.9 (0.4)	54.0	52–54
Brixton	28.9 (12.4)	28.0	4–52	22.6 (8.8)	20.0	7–40
Trails B	181.9 (85.4)	178.0	44.2–300	87.4 (33.7)	83.8	33–160
Trails A	91.9 (60.6)	75.0	20–300	36.6 (9.8)	35.6	17.5–63.7
Digits backwards	3.3 (2.9)	4.0	0–11	7.4 (2.6)	7.0	2–14
Digits forwards	5.8 (4.1)	6.0	0–14	10.2 (2.2)	10.0	6–14
HVLT (RDI)	6.0 (3.9)	7.0	0–12	9.7 (1.8)	10.0	6–12
HVLT (delayed)	3.6 (2.9)	3.5	0–9	7.3 (2.8)	7.0	3–12
HVLT (total)	11.6 (7.8)	13.0	0–23	23.3 (4.9)	23.5	13–32
Rey Complex Figure (delay)	10.0 (9.2)	8.5	0–30	18.2 (6.6)	17.8	7–32
Rey Complex Figure (immediate)	11.2 (9.0)	9.0	0–29	19.5 (6.6)	19.3	7.5–32
Rey Complex Figure (copy)	23.9 (11.7)	26.0	0–36	34.5 (2)	35.0	28.5–38
Animal fluency	10.2 (7.5)	10.5	0–25	24.7 (7)	24.0	16–44

*HVLT, Hopkins Verbal Learning Test; RDI, recognition discrimination index*.

**Figure 2:**
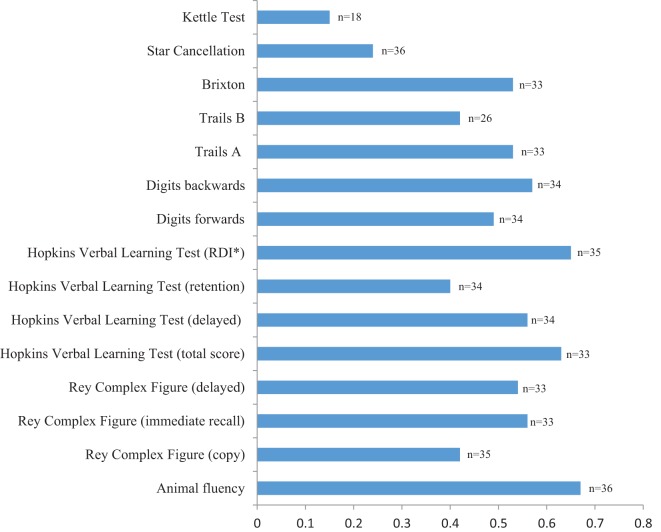
**Association between auditory comprehension and the cognitive tests (*R*^2^), with demographic factors included in the models**. *RDI, recognition discrimination index.

Figure 2 shows that all cognitive tests were significantly associated with auditory comprehension (all *p* < 0.01) with a variance ranging from 40 to 67%, except for the Kettle Test [*F*(3,14) = 0.75, *p* = 0.54] with a variance of 14%, and the Star Cancellation [*F*(3) = 4.9, *p* = 0.18] with a variance of 24%. A multiple logistic regression was used for Star Cancellation due to a ceiling effect (refer to Table 2), and a pseudo *R*^2^ was reported. Animal fluency had the highest variance explained by auditory comprehension (67%), closely followed by HVLT RDI (65%) and immediate recall (63%).

**Figure 3:**
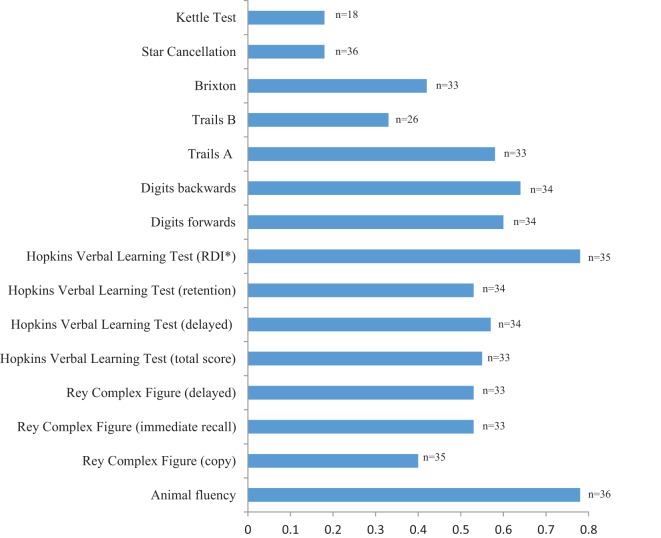
**Association between naming and the cognitive tests (*R*^2^), with demographic factors included in the models**. *RDI, recognition discrimination index.

Figure 3 displays the results of the multiple linear regressions used to determine the relationship between the naming and the cognitive tests, with age and education included in the models. A multiple logistic regression was again used for the Star Cancellation Test. All cognitive tests were significantly associated with naming (all *p* < 0.01) with a variance ranging from 33 to 78%, except for the Kettle Test [*F*(1,16) = 3.44, *p* = 0.08] with a variance of 18%, and the Star Cancellation [*F*(3) = 3.8, *p* = 0.28] with a variance of 18%. Animal fluency and the HVLT RDI had the highest variance explained by naming (both 78%).

## Discussion

Both auditory comprehension and naming performance in aphasia were significantly associated with all pen-and-paper cognitive tests, with the lone exception of Star Cancellation. The total variance explained by auditory comprehension performance differed between the cognitive tests. The cognitive tests requiring a verbal response showed more variance explained by naming compared to the non-verbal cognitive tests. We also confirmed that auditory comprehension and naming were not significantly associated with an everyday real-life measure of cognition (Kettle Test). Feasibility was an issue, with substantial missing data for the pen-and-paper cognitive tests, and also missing data for the Kettle Test due to upper and lower limb hemiparesis, in aphasia. While non-linguistic cognitive impairments co-occur with aphasia (2–4), non-verbal cognitive tests may not necessarily overcome the potential confounding influence of aphasia-related deficits. The Kettle Test shows that individuals with aphasia can undertake a real-life cognitive task without the confounding influence of language impairments.

Animal fluency and the HVLT RDI had the highest variance explained by both auditory comprehension and naming. Our animal fluency results are supported by Whiteside et al. (18) where factor analysis was used to verify that animal fluency loaded exclusively to language, rather than executive functioning. Although executive skills may be impaired in aphasia (38) using the animal fluency task to determine executive skills in people with aphasia may mislead diagnoses.

The RDI component of the HVLT requires a yes/no response to identify previously learned words. Eliciting a yes/no response from a person with aphasia is a suggested technique to overcome verbal barriers and facilitate communication (39), yet the variance was largely explained by auditory comprehension (65%) and naming (78%). These results may not be surprising given the HVLT requires participants to remember linguistic targets, thus impaired language will influence recognition performance. Also, to identify a correct response, participants need to discriminate between semantically related distractors. The literature supports observed semantic deficits in both auditory comprehension and naming in aphasia (40). Thus, using semantically related distractors in a verbal recognition task will likely be confounded in aphasia, even when the response is restricted to a yes/no response.

The total variance explained by auditory comprehension for the pen-and-paper cognitive tests without language stimuli or a verbal response (i.e., Star Cancellation, Rey Complex Figure, and the Brixton) was variable (24–56%). This means a large amount of variance remains unexplained, which may be attributed to concomitant non-linguistic cognitive deficits. Auditory comprehension was not significantly associated with the Star Cancellation Test. A weak association between neglect and language comprehension stroke is verified in the literature (41), but the simplicity of the Star Cancellation’s instructions, and the simplicity of the response (crossing out stars with a pen), will assist comprehension in aphasia. The Star Cancellation Test was able to be completed by all participants with aphasia, and it is a reliable assessment to use poststroke (30) where visual spatial screening is recommended.

There was a significant association between all subtests of the Rey and auditory comprehension. Pyun et al. (42) explored visuospatial skills in 23 participants with aphasia and found that the Rey copy scores were significantly correlated with the severity of the overall language performance (*r* = 0.654, *p* < 0.05). Visual perceptual deficits may be underestimated in aphasia. While the Rey copy is supported by simple verbal instructions, the complex copy task has been shown to involve planning and organization skills for successful completion (43). Thus, the relationship with language performance and the Rey copy task could be partly explained by concomitant executive deficits in aphasia. The association between non-linguistic memory performances in the Rey immediate task can be compared with Lang and Quitz (44), where 99 participants poststroke (49 with aphasia and 50 without aphasia) were assessed using linguistic and non-linguistic memory tests. Participants with aphasia performed worse than participants without aphasia in the memory tests, even when participants had similar cerebral lesions, which the authors attributed to a common working memory impairment in aphasia.

The total variance explained by auditory comprehension for the Brixton was 53%. The aphasia group, and to a lesser degree the controls, experienced difficulty understanding the Brixton’s lengthy verbal instructions. This was evidenced by the need to repeat instructions for clarity. However, as part of the Brixton assessment, direct feedback is provided for each response (e.g., participants are aware of a correct or incorrect response based on where the blue dot appears on the following sheet). This immediate visual feedback may have assisted with participants learning what is needed. Thus, executive tests that necessitate lengthy verbal instructions can incorporate non-linguistic prompts to facilitate understanding.

Fucetola et al. (21) explored the association between auditory comprehension and non-verbal subtests of the WAIS-III and WMS-III [e.g., block design (constructional), matrix reasoning (reasoning by visual analogy), picture arrangement (sequencing), and spatial span (visual working memory)]. Auditory comprehension accounted for 41% of the total variance in the non-verbal cognitive tests. Naming was also significantly associated with the non-verbal cognitive tests in the present study, which contrasts with the findings of Fucetola et al. (21). It is difficult to distinguish between a confounding language influence and a co-occurring non-linguistic cognitive impairment in cognitive tests that are not tailored for individuals with aphasia.

Auditory comprehension was not significantly associated with the Kettle Test. This everyday real-life cognitive test contains verbal instructions, but understanding is maximized by using a meaningful task with familiar everyday objects. The kitchen setting may further support understanding by incorporating a multisensory environment. Using multiple sensory modalities facilitates the ability to identify, discriminate, and recognize stimuli, and learning can be optimized (45, 46). Our results demonstrate that using a familiar, real-life functional measure of cognitive performance may minimize the language skills needed to complete the task. The Kettle Test may be appropriate for individual with aphasia, but participants needed adequate motor skills to complete the task. Upper and lower limb hemiparesis was the sole reason for missing data associated with the Kettle Test. While the Kettle Test is regarded as an executive task (37), it may underestimate the potential association between language and cognitive skills needed for more complex community living activities. Further testing using functional cognitive performance measures in aphasia is needed.

Testing cognition in aphasia was not feasible in a number of participants, particularly those with more severe language impairments. There were no missing data for the language tests in both the aphasia and control group. Primary reasons for missing data in the pen-and-paper cognitive tests were participant refusal and an inability to understand the tasks. Chapman (47) explored the association between semantic comprehension deficits and executive skills in aphasia and semantic dementia and reported that participants found many executive tests too difficult to understand. If an individual is unable to undertake task instructions, performance may reflect comprehension deficits rather than the target non-linguistic cognitive domain intended for testing. This may result in inaccurate information being used to guide cognitive therapy, inaccurate education given to stroke survivors and their families, and the potential for misinformed discharge planning. Missing data associated with the Kettle Test were due to upper and lower limb hemiparesis. Participants with aphasia were particularly resistant to participate in the Trail Making Test (part B). This executive task has linguistic stimuli and requires a more complex response (i.e., participants use a pen to sequentially track the alternate numbers and letters). In contrast to another executive task, the Brixton, a simple response is required (i.e., pointing to a colored circle), and participants were more likely to attempt and complete it. It appears that feasibility of testing participants with aphasia not only relates to complexity of instructions but it may also be influenced by the complexity of the response needed for completion.

To determine feasibility of cognitive testing, we minimized the exclusion criteria to be inclusive of participants that represent clinical practice. A limitation is that the high frequency of missing data for the cognitive tests may have biased the regression findings to exclude the association of participants with profound comprehension deficits and cognitive performance.

Assessing non-linguistic cognitive skills in aphasia is challenging, which results in people with aphasia being excluded from studies that have validated cognitive assessments in stroke (10). Using non-verbal cognitive tests may not ensure accurate results due to potentially confounding auditory comprehension impairments observed in aphasia. Difficulty understanding the tasks may also influence an individual’s willingness to participate in testing, creating feasibility barriers for both clinical and research practice. Clinical guidelines for poststroke aphasia (48, 49) require further evidence of the association between linguistic and non-linguistic cognitive skills in aphasia, to warrant the inclusion of non-linguistic cognitive assessment in clinical recommendations. The Star Cancellation Test and the Kettle Test were the only cognitive assessments not significantly associated with auditory comprehension and naming performance in aphasia. To maximize the accuracy and feasibility of cognitive testing in aphasia, cognitive tests need to be tailored to enhance understanding of the tasks. Multidisciplinary expertise is needed to look beyond typical pen-and-paper methods and consider multisensory input for cognitive testing in aphasia.

## Ethics Statement

Ethical clearance was obtained through relevant Human Research Ethics Committees in Brisbane, Australia, including the Royal Brisbane and Women’s Hospital. Written consent was sourced for all participants and a substitute decision maker was used for patients with cognitive deficits that precluded informed consent.

## Author Contributions

KW, TC, and DC contributed to the conception and design of the work. KW was responsible for data collection, data analysis, and drafting the manuscript. TC and DC critically revised the work and contributed to the interpretation of the data. All authors gave their final approval of the version to be published and agreed to be accountable for all aspects of the work.

## Conflict of Interest Statement

The authors declare that the research was conducted in the absence of any commercial or financial relationships that could be construed as a potential conflict of interest.
